# The Role of Gut Microbiota in Anxiety, Depression, and Other Mental Disorders as Well as the Protective Effects of Dietary Components

**DOI:** 10.3390/nu15143258

**Published:** 2023-07-23

**Authors:** Ruo-Gu Xiong, Jiahui Li, Jin Cheng, Dan-Dan Zhou, Si-Xia Wu, Si-Yu Huang, Adila Saimaiti, Zhi-Jun Yang, Ren-You Gan, Hua-Bin Li

**Affiliations:** 1 School of Public Health, Sun Yat-sen University, Guangzhou 510080, China; xiongrg@mail2.sysu.edu.cn (R.-G.X.); chengj225@mail2.sysu.edu.cn (J.C.); zhoudd6@mail2.sysu.edu.cn (D.-D.Z.); wusx6@mail2.sysu.edu.cn (S.-X.W.); huangsy9@mail2.sysu.edu.cn (S.-Y.H.); saimaiti@mail2.sysu.edu.cn (A.S.); yangzhj57@mail2.sysu.edu.cn (Z.-J.Y.); 2 School of Chinese Medicine, Li Ka Shing Faculty of Medicine, The University of Hong Kong, Hong Kong 999077, China; lijiahui@hku.hk; 3 Singapore Institute of Food and Biotechnology Innovation (SIFBI), Agency for Science, Technology and Research (A*STAR), 31 Biopolis Way, Singapore 138669, Singapore

**Keywords:** anxiety, depression, gut microbiota, probiotics, natural products

## Abstract

The number of individuals experiencing mental disorders (e.g., anxiety and depression) has significantly risen in recent years. Therefore, it is essential to seek prevention and treatment strategies for mental disorders. Several gut microbiota, especially Firmicutes and Bacteroidetes, are demonstrated to affect mental health through microbiota–gut–brain axis, and the gut microbiota dysbiosis can be related to mental disorders, such as anxiety, depression, and other mental disorders. On the other hand, dietary components, including probiotics (e.g., *Lactobacillus* and *Bifidobacterium*), prebiotics (e.g., dietary fiber and alpha-lactalbumin), synbiotics, postbiotics (e.g., short-chain fatty acids), dairy products, spices (e.g., *Zanthoxylum bungeanum*, curcumin, and capsaicin), fruits, vegetables, medicinal herbs, and so on, could exert protective effects against mental disorders by enhancing beneficial gut microbiota while suppressing harmful ones. In this paper, the mental disorder-associated gut microbiota are summarized. In addition, the protective effects of dietary components on mental health through targeting the gut microbiota are discussed. This paper can be helpful to develop some dietary natural products into pharmaceuticals and functional foods to prevent and treat mental disorders.

## 1. Introduction

Mental health is one of the United Nations’ Sustainable Development Goals, and mental disorders mainly include anxiety, depression, bipolar disorder, autism spectrum disorder (ASD), schizophrenia, and eating disorders [1]. In 2019, the number of individuals suffering from mental disorders was estimated to be close to 970 million [2]. Mental disorders can influence the study, work, and normal life of patients, and lead to suicide in severe situations. Moreover, mental disorders can affect the normal life of family members of the patients. Mental disorders have emerged as a significant public health concern worldwide, and also lead to a huge medical burden and economic loss. The COVID-19 pandemic resulted in quarantine, economic decline, unemployment, etc., which have led to a marked increase in mental health problems [3,4]. Although the pandemic has gradually passed, recovery of the economy to a normal level will take a long time, and the condition of lower income and unemployment will continue. The influences of the pandemic on mental health, especially anxiety, depression, and posttraumatic stress disorder (PTSD), will last a very long time. On the other hand, the gut microbiota and its metabolites have a significant influence on preserving the overall health of the host; gut microbiota dysbiosis has been reported to be associated with the occurrence and development of several chronic metabolic diseases, such as obesity, diabetes mellitus, and cancers [5,6,7,8,9]. Moreover, gut microbiota can also be correlated with mental health, which has received increasing attention in recent years [10,11,12,13,14]. It was reported that the gut microbiota could influence the brain and mental health in several ways, such as the vagus nerve, microbial regulation of neuro-immune signaling, microbiota-mediated tryptophan metabolism, microbial control of neuroendocrine function, and microbial production of neuroactive compounds [15,16]. In addition, the gut microbiota could produce and regulate neurotransmitters, such as serotonin, dopamine, and glutamate, which play important roles in neurological and immunological activities in the brain [17]. Moreover, a multiomics study based on the “Lunar Palace 365” experiment found *Bacteroides uniformis*, *Roseburia inulinivorans*, *Eubacterium rectale*, and *Faecalibacterium prausnitzii* exerted a positive effect on the maintenance of mental health by producing short-chain fatty acids (SCFAs) and regulating amino acid, taurine, and cortisol metabolism pathways [18]. Additionally, gut microbiota dysbiosis could also promote the occurrence and progression of mental disorders [17,19]. Therefore, it could be a potential method to target gut microbiota for the prevention and treatment of mental disorders. At present, the main treatments for mental disorders include pharmacotherapy and psychotherapy, which are easily interrupted and have limited effectiveness, and some drugs might cause side effects [20,21]. Therefore, other preventive and treatment methods, such as acupuncture, meditation, and natural products, have also attracted increasing attention [20]. The effects of natural products against mental disorders have become a research hotspot in the fields of food science, nutrition, psychology, and psychiatry in recent years [16,22,23,24,25]. Furthermore, the studies showed that some probiotics and natural products exerted vital roles in the management of mental disorders via modulating gut microbiota [15,26,27]. For example, a study found that high intakes of vegetables, fruits, and fiber were positively associated with mental health in a population of 502,494 middle-aged adults [28]. Another study based on 482 participants showed that the tryptophan-rich diet was negatively correlated with depression and could improve social cognition [29].

In this narrative review, we conducted a comprehensive search of Web of Science Core Collection and PubMed databases, gathered relevant high-quality literature published within the past five years, and retrieved the keywords of anxiety, depression, bipolar disorder, autism spectrum disorder, schizophrenia, mental disorder, mental health, gut microbiota, probiotics, prebiotics, postbiotics, dairy product, spice, fruit, vegetable, medicinal herb, and natural product. In this review paper, the relationships between gut microbiota and mental disorders are first summarized, followed by a discussion of the impacts of natural dietary products on mental health by regulating the gut microbiome, emphasizing the underlying mechanisms. This review paper could be helpful for people to make informed choices regarding natural dietary products for the prevention and management of mental disorders, and it may also promote the development of natural dietary products by the industry as pharmaceuticals and functional foods to maintain mental health. 

## 2. Gut Microbiota and Mental Disorders

The composition of gut microbiota is complex; some microorganisms may protect mental health, while others may be related to the onset and development of mental disorders. In the following part, the association of gut microbiota with certain mental disorders is summarized below, and more detailed information can be found in Table 1 and Table 2.

### 2.1. Anxiety

Anxiety is among the most common mental disorders [61]. It is indicated that certain gut microbiota are correlated with anxiety. For example, social exclusion is one of the causes of anxiety. A study found that the abundance of *Prevotella* was increased, while the *Firmicutes*/*Bacteroidetes* ratio and the abundance of *Faecalibacterium* spp. were significantly reduced in individuals with social exclusion [30]. Moreover, a study of 198 Spanish individuals found that patients with anxiety had lower Simpson’s diversity [57]. Additionally, patients with generalized anxiety disorder (GAD) had lower microbial richness and diversity, as well as reduced levels of *Firmicutes* spp. and microbiota that produce SCFAs, but more *Fusobacteria* and *Bacteroidetes* [32]. In addition, a prospective observational study showed that ulcerative colitis patients with anxiety exhibited a reduction in fecal microbiome richness and diversity, the abundances of *Prevotella_9* and *Lachnospira*, as well as immunoglobulin proteins, but had an increase in the abundances of *Lactobacillales*, *Sellimonas*, *Streptococcus*, and *Enterococcus* [31]. Moreover, a study showed that the fecal microbiome could influence anxiety-related behavior in mice [62]. Another study indicated that mice with higher anxiety had significantly lower levels of *Firmicutes* [58].

In brief, patients/mice with anxiety showed dramatically decreased microbial richness and diversity. At the phylum level, anxiety patients/mice usually had lower *Firmicutes*, but higher *Bacteroidetes* and *Fusobacteria*. At the genus level, several gut microbiota genera were positively correlated with anxiety, such as *Prevotella*, *Lactobacillales*, *Sellimonas*, *Streptococcus*, and *Enterococcus*, while some gut microbiota genera were inversely correlated with anxiety, suggesting that targeting these gut microbiomes could be a promising approach for preventing anxiety. At present, most studies focus on the genus level of anxiety-related gut microbiota. In the future, more research should highlight the species level of gut microbiota, considering that different species in the same genera could exert different functions, or even the opposite functions, on anxiety.

### 2.2. Depression

Depression is considered a major public health problem [63]. Depression can lead to several serious outcomes and relates to a high suicide rate. Studies showed that gut microbiome dysbiosis was associated with the occurrence and development of depression [64,65]. A study found obvious differences of fecal microbiota composition in four phyla as well as in the abundances of 16 bacterial families between healthy individuals and major depressive disorder (MDD) patients [36]. Another study found a relative reduction in the alpha diversity of gut microbiota in patients with current depressive episodes [33]. Additionally, a study based on a large microbiome population cohort showed that *Dialister* and *Coprococcus* spp. were decreased in patients with depression [34]. Furthermore, the evidence showed that MDD patients had higher levels of *Prevotella*, *Klebsiella*, *Streptococcus* and *Clostridium* XI, but lower levels of *Bacteroidetes* [35]. Moreover, *Helicobacter pylori* (*H. pylori*) infection could induce a pathological state of gastrointestinal flora. For instance, a cross-sectional study including 5558 Chinese people found a significantly higher risk of depressive symptoms in women infected with *H. pylori*, but not in men [66]. This could be attributed to women who were more likely to feel anxious and depressed compared with men due to the relationship between *H. pylori* and cancer [67]. In addition, premenopausal women with depression had higher levels of estradiol-degrading bacteria (*Klebsiella aerogenes*) compared to healthy controls [37]. Furthermore, it was reported that fecal transfers from patients with depression to germ-free-like mice could induce depressive-like behaviors [68]. Another study found that depressive macaques had higher abundances of six gut bacteria species mainly from the Paraprevotella family, but had lower abundances of another eight gut bacteria species mainly from the Streptococcaceae and Gemella families [59]. Another study demonstrated that gut microbiota could affect the expression of proteins in several tissues related to the gut–brain axis, thereby contributing to the development of depression [69]. Additionally, the transplantation of fecal microbiota from healthy Sprague–Dawley rats could prevent the development of depression in Fawn-hooded rats by significantly decreasing several gut microbial species, such as *Dialister* sp., which could modulate the immune and metabolic activity of the host.

In a word, gut microbiota in individuals/animals with depression were found to differ in composition and abundance from those in healthy controls. At the family level, certain gut microbiota were positively correlated with depression, such as Paraprevotella, but some others were negatively associated with depression, such as Streptococcaceae and Gemella. At the genus level, several gut microbiota genera, such as *Prevotella*, *Klebsiella*, and *Clostridium*, showed a positive relationship with depression. Moreover, gut microbiota dysbiosis might be a crucial factor in the pathogenesis of depression via influencing the protein expression in tissues related to the gut–brain axis. Although the specific gut microbiota of people with depression varied from study to study, all these discoveries showed that gut microbiota composition significantly changed in depressive individuals and indicated that gut microbiota might be a novel target for the prevention and management of depression. Similar to the anxiety mentioned above, most of the studies about gut microbiota related to depression have been mainly at the genus level in recent years. More research studies should be conducted at the species level to further investigate the connection between gut microbiota and depression, since the effects of different species of gut microbiota in the same genera on depression could be different or opposite. 

### 2.3. Bipolar Disorder

Bipolar disorder is a chronic and incapacitating illness that often reoccurs, leading to cognitive and functional impairment [70]. Several compelling lines of evidence linked the gut microbiome to bipolar disorder. For instance, a study showed that bipolar disorder patients had a reduction in gut microbiota diversity, with more *Clostridiaceae* and *Collinsella* [38]. Additionally, a cross-sectional study indicated that *Flavonifractor* was associated with bipolar disorder (odds ratio (OR), 2.9; 95% CI, 1.6–5.2) [41]. Another cross-sectional study found that *Faecalibacterium* significantly decreased in bipolar disorder patients [39]. Moreover, it was reported that bipolar disorder patients had more Actinobacteria and Coriobacteria, but less Ruminococcaceae and Faecalibacterium [40]. However, there was no significant difference in either *Bifidobacterium* (phylum Actinobacteria) or *Lactobacillus* bacterial counts between the two groups of 39 bipolar disorder patients and 58 healthy individuals [71]. The discrepancy among these studies might be caused by the differences in the characteristics of the participants. 

In general, bipolar disorder patients had higher abundances of *Clostridiaceae*, *Collinsella*, and the phyla of Actinobacteria and Coriobacteria, but lower abundances of Faecalibacterium and Ruminococcaceae. Further large-scale population studies are required in the future to investigate the impact of various gut microbiota on bipolar disorder, and the participants should be more representative, such as more races from different countries with different gender as well as age. 

### 2.4. Autism Spectrum Disorder

ASD is a heterogeneous neurodevelopmental disorder [72]. Research has shown that the composition and abundance of gut microbiota differ between individuals with ASD and those without the disorder. For example, a study found that children with Pitt–Hopkins syndrome (a severe ASD) had a higher relative abundance of *Clostridium bolteae* than their unaffected family members [42]. Another study indicated that higher levels of *Clostridium paraputri*, *Clostridium bolteae*, and *Clostridium perfringens* were found in the feces of Egyptian ASD children. In addition, *Clostridium diffiicile* and *Clostridium clostridiioforme* were only found in ASD children, while *Clostridium tertium* was only found in normal children [44]. Moreover, a cross-sectional case-control study found that ASD children had higher abundances of *Actinobacteria*, *Proteobacteria*, as well as *Bacilli* [45]. Additionally, it was demonstrated that *Fmrl* KO mice with autistic-like behaviors had a reduced population of *Akkermansia muciniphila*, accompanied with increased levels of TNF-α and Iba1 [60]. Furthermore, the gut microbiome might also be linked to the severity of ASD. For instance, it was reported that ASD children with a sleep disorder had higher severity of core symptoms of ASD and lower abundances of *Faecalibacterium* and *Agathobacter*. These bacterial strains were positively linked to the levels of 3-hydroxybutyric acid and melatonin, but negatively correlated with the level of serotonin [43]. 

Overall, several species of gut microbiota were only found in ASD patients, such as *Clostridium diffiicile* and *Clostridium clostridiioforme*. Moreover, certain gut microbiota were increased in ASD patients/animals, such as *Actinobacteria*, *Proteobacteria*, and *Bacilli*, whereas some were decreased, such as *Akkermansia muciniphila*, *Faecalibacterium* and *Agathobacter*. In the future, more studies about the association between gut microbiota and ASD need to be conducted to find out more beneficial or harmful gut microbiota, which could be targeted for the management of ASD.

### 2.5. Schizophrenia

Schizophrenia affects 1% of the world’s population, and patients with schizophrenia may exhibit positive symptoms like hallucinations and disorganized speech, negative symptoms like an absence of interest and motivation, and cognitive deficits like impaired executive functions and memory [73]. 

Mounting evidence indicated that schizophrenia was associated with gut microbiota dysbiosis. For instance, a case-control study showed that gut microbiome dysbiosis was found in schizophrenia patients [74]. A cross-sectional study found that several gut bacteria could only be found in healthy participants, but were missing in patients with schizophrenia, such as *Haemophilus* [46]. However, another study found a positive correlation between the level *Haemophilus* abundance and negative symptoms of schizophrenia [47]. The discrepancy between the two studies might be associated with the participants having different types of schizophrenia. Moreover, it was reported that schizophrenia patients showed reduced abundances of *Ruminococcus* and *Roseburia*, as well as an increased abundance of *Veillonella* [48]. Another study demonstrated that schizophrenia patients had a different gut microbial composition, as well as a higher abundance of *Lachnospiraceae* [49]. Additionally, a study based on 90 medication-free schizophrenia patients and 81 controls showed that several facultative anaerobes, which were rare in healthy individuals, were found in schizophrenic patients, such as *Lactobacillus fermentum* and *Enterococcus faecium* [52]. Another cross-sectional study indicated that patients with schizophrenia exhibited a higher abundance of *Proteobacteria*, but lower abundances of *Faecalibacterium* and *Lachnospiraceae* [51]. In addition, a study based on 82 schizophrenia patients and 80 controls showed that the abundance of *Succinivibrio* was positively correlated with the severity of schizophrenia symptoms, while the abundance of *Corynebacterium* was negatively related to the negative symptoms [50]. Additionally, several other bacterial families were proven to be correlated to the severity of schizophrenia, such as Veillonellaceae and Lachnospiraceae. The gut microbiota could alter neurochemistry and neurologic function via modulating the glutamate–glutamine–γ-aminobutyric acid (GABA) cycle [75].

Collectively, some special gut bacteria, such as *Lactobacillus fermentum*, *Enterococcus faecium*, and *Alkaliphilus oremlandii*, could be only found in patients with schizophrenia. Some gut microbiota were positively correlated to the severity of schizophrenia, such as *Lachnospiraceae*, *Veillonella*, *Collinsella*, *Lactobacillus*, *Succinivibrio*, and *Corynebacterium*, whereas some were negatively correlated with schizophrenia, such as *Coprococcus*, *Ruminococcus*, *Roseburia*, *Adlercreutzia*, *Anaerostipes*, and *Faecalibacterium*. Furthermore, the gut microbiota could affect neurochemistry and neurologic function through modulating the metabolism of enteric and central nervous system function-related molecules, such as GABA. A supplement of beneficial gut microbiota, which were absent in patients with schizophrenia, might be useful for the treatment of schizophrenia. Alternatively, the reduction in harmful gut microbiota, which were only found in patients with schizophrenia, could be another therapeutic strategy via the administration of medicine or functional foods. 

### 2.6. Other Mental Disorders

In addition to the mental disorders mentioned above, many other mental disorders are linked to the gut microbiota, such as anorexia nervosa, PTSD, and attention-deficit/hyperactivity disorder (ADHD). For example, it was reported that patients with anorexia nervosa had a reduction in Bacteroidetes and gut microbiota dysbiosis, contributing to anorexia nervosa-specific pathologies [76]. Another study based on both humans and mice found that gut microbiota dysbiosis contributed to the pathogenesis of anorexia nervosa [53]. Moreover, a study showed that the uncultured *Eubacterium hallii* and *Bacteroides eggerthii* were correlated to the reappearance of post-traumatic stress symptoms in frontline healthcare workers during the COVID-19 pandemic [54]. Another study found that individuals with PTSD had higher abundances of *Mitsuokella*, *Odoribacter*, *Catenibacterium*, and *Olsenella* [55]. A study of 198 Spanish individuals found that patients with comorbid symptoms of PTSD, depression, and trait anxiety had lower levels of *Fusicatenibacter saccharivorans* [57]. Additionally, ADHD children exhibited decreased relative abundances of genera *Agathobacter*, *Anaerostipes*, and *Lachnospiraceae*, as well as the plasma level of TNF-α [56]. Another study included 95 participants found that the gut microbiota compositions of individuals with ADHD and ASD were highly similar in terms of both alpha- and beta-diversity, and had an increased concentration of lipopolysaccharide-binding protein, which was positively correlated with IL-8, IL-12, and IL-13 [77].

**Figure 1:**
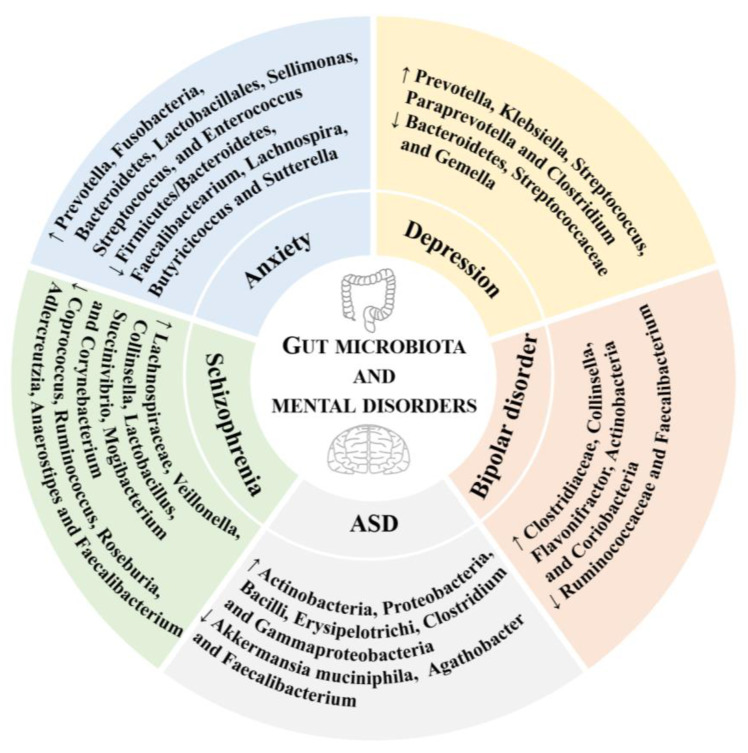
The relationships of gut microbiota and mental disorders. ↑ represents positive association, ↓ represents negative association. ASD, autism spectrum disorder.

In short, some gut microbiota were associated with some mental disorders, such as anxiety, depression, bipolar disorder, ASD, schizophrenia, anorexia nervosa, PTSD, and ADHD (Figure 1), which could be potential targets for the prevention and treatment of these mental disorders. Moreover, further high-quality studies are required to explore the effects of various gut microbiota on different mental disorders.

## 3. Effects and Mechanisms of Dietary Components on Mental Disorders through Modulating Gut Microbiota

**Figure 2:**
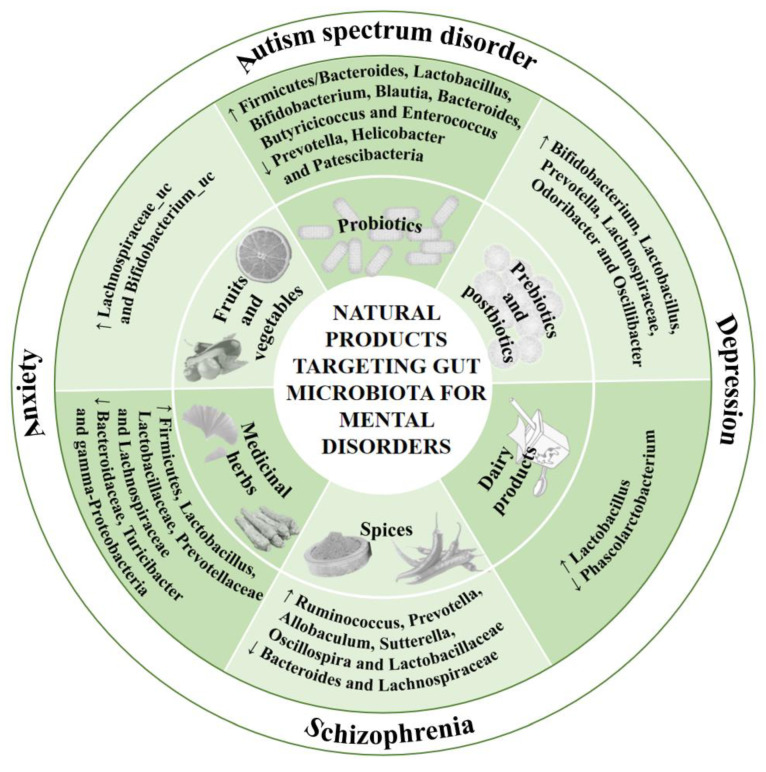
The effects of natural products on mental disorders. ↑ represents increase, ↓ represents decrease.

Many dietary components have been shown to exert protective effects against mental disorders through regulating gut microbiota, such as probiotics, prebiotics, postbiotics, fruits, vegetables, and spices, which are discussed below and are shown in Figure 2 and Table 3 and Table 4.

### 3.1. Probiotics

Probiotics have become a hotspot in the research fields of foods, nutrition, biology, and medicine, and can be used to prevent and manage several diseases, such as constipation, obesity, and cardiovascular disease. As mentioned above, several mental disorders were associated with abnormalities in gut microbiota. An increasing number of studies have revealed that probiotics, particularly the genus *Lactobacillus*, could prevent and manage several mental disorders by modulating the gut microbiota. For instance, a study showed that *Lactobacillus murine* (*L. murine*) and *L. reuteri* could increase GABA content in the hippocampus and alleviate depression-like behaviors in Dcf1 knockout mice [110]. Moreover, *L. rhamnosus* zz-1 alleviated depression-like behaviors in mice induced by chronic unpredictable mild stress (CUMS) improved hypothalamic–pituitary–adrenal (HPA) axis hyperactivity, and increased monoamine neurotransmitters, brain-derived neurotrophic factor (BDNF), and tyrosine kinase receptor B (TrkB) through regulation of gut microbiota, such as recovering the relative abundances of *Lachnospiraceae* NK4A136, *Bacteroides* as well as *Muribaculum* [79]. Additionally, it was reported that probiotic *Pediococcus acidilactici* CCFM6432 could alleviate anxiety-like behaviors caused by stress through inhibiting the over-proliferation of *Escherichia shigella* and promoting *Bifidobacterium* growth in C57BL/6 mice [83]. Moreover, *Akkermansia muciniphila* reduced depressive-like behavior in mice through reversing gut microbial abnormalities [84]. In addition, heat-killed *Enterococcus faecalis* strain EC-12 reduced anxiety-like behavior and enhanced *Butyricicoccus* and *Enterococcus* in mice [80]. A study based on 423 women in New Zealand showed that *L. rhamnosus* HN001 significantly decreased the depression and anxiety scores of women in the postpartum period [111]. Another double-blind RCT showed that *L. rhamnosus* Probio-M9 enhanced the psychological and physiological qualities of life in stressed adults through increasing the relative abundances of certain species of gut microbiota [98]. 

The multi-strain probiotic formulation could also exert preventive and therapeutic effects on mental disorders. A study based on 156 adults with subclinical symptoms of mental disorders showed that the mixture of *Lactobacillus reuteri* NK33 and *Bifidobacterium adolescentis* NK98 improved mental health and sleep through modulating gut microbiota [99]. Another multi-strain probiotic plus biotin treatment showed a beneficial effect on depression, and increased beta-diversity as well as abundances of *Ruminococcus gauvreauii* and *Coprococcus 3* [102]. Moreover, the proportions of *Actinobacteria*, *Cyanobacteria*, and S24-7_unclassified were decreased in the gut of stress mice, while multi-strains of probiotics recovered these changes as well as reduced depressive-like behaviors in mice [81]. 

In short, probiotics, such as *Lactobacillus*, *Bifidobacterium*, *Pediococcus acidilactici CCFM6432*, and *Akkermansia muciniphila*, showed significant preventive and therapeutic effects on several mental disorders, such as anxiety and depression. They influenced neurotransmitter metabolism, improved HPA axis hyperactivity, and increased the expression of BDNF and TrkB through modulating gut microbiota. Furthermore, multi-strain probiotic formulations might have more efficient actions on mental health protection compared with a single probiotic strain. It is also important to consider the interactions between different bacteria and their metabolites when exploring multi-strain probiotics formulations. The beneficial effects of more probiotics on different mental disorders (not only anxiety and depression) should be investigated by high-quality clinical trials in the future. 

### 3.2. Prebiotics and Postbiotics

The studies indicated that prebiotics and postbiotics could also be potential for the prevention and management of mental disorders, such as anxiety, depression, ASD, and schizophrenia [112,113,114]. 

It was reported that dietary fibers could improve the relationship between gut microbiota and the central nervous system in schizophrenia patients through regulating gut microbiota [113]. A double-blind RCT found that the galacto-oligosaccharides (GOS) prebiotic could alleviate anxiety and upregulate the abundance of *Bifidobacterium* in the 4-week intervention of 64 late adolescent females [103]. Another study showed that the 6-week administration of Bimuno^®^ galactooligosaccharide (B-GOS^®^) prebiotic dramatically increased *Lachnospiraceae* and enhanced anti-social behavior in 30 autistic children [105]. However, administration of the prebiotic 4G-beta-D-galactosucrose (LS) did not improve depressive symptoms or the abundance of *Bifidobacterium* in a study based on 20 depression patients in Japan [106]. The discrepancy between these results might be due to the differences in race, prebiotic types, and so on. Furthermore, the combination of probiotics and prebiotics (synbiotics) exerted protective effects on mental health. The study showed that the 4-week administration of a supplement containing probiotics, prebiotics, plant extracts, and nutrients exerted positive influences on mental health via increasing the abundances of *Lactobacillus* and *Bifidobacterium* [104]. Additionally, synbiotics could also alleviate the side effects caused by antipsychotics. It was found that the synbiotics attenuated olanzapine-induced weight gain and insulin resistance in schizophrenia patients [115]. 

A study showed that SCFAs could ameliorate high fructose-induced depressive-like behaviors in mice by improving hippocampal neurogenesis decline and blood–brain barrier damage [116]. It was found that butylated starch alleviated chronic restraint stress-induced depression-like behaviors and excessive corticosterone production in mice by regulating the gut microbiota [86]. Additionally, the combination of prebiotics and postbiotics could exert synergistic effects on several mental disorders. For instance, a study showed that the alpha-lactalbumin and sodium butyrate improved some pathological aspects of mice behaviors relevant to autism and depression either alone or in combination, and the combination was more effective [117]. 

In a word, prebiotics (e.g., dietary fiber, GOS, B-GOS^®^ and alpha-lactalbumin) as well as postbiotics (such as SCFAs) exerted protective effects on mental health, especially in combinative administration. Moreover, prebiotics could be a potential agent to alleviate the side effects of antipsychotics. In the future, the effects of more prebiotics and postbiotics on different mental disorders should be studied. Because synbiotics could exert the synergistic or additive effects of probiotics and prebiotics, more attention should be paid to the effects of synbiotics on mental disorders. In addition, because postbiotics are considered to be safer and more stable than probiotics, their effects on mental disorders should also be highlighted. Furthermore, more large-scale clinical trials are necessary to prove the influences of prebiotics and postbiotics on mental disorders, including health benefits as well as side effects.

### 3.3. Dairy Products

Dairy products are important components of both traditional and modern diets, and have a variety of health benefits. A double-blinded RCT based on 82 depressive patients with constipation found that the intervention of a fermented dairy beverage containing *Lacticaseibacillus paracasei* strain Shirota for 9 weeks increased beneficial bacteria (e.g., *Adlercreutzia*, *Megasphaera* and *Veillonella*), but decreased harmful bacteria (e.g., *Rikenellaceae_RC9_gut_group*, *Sutterella* and *Oscillibacter*) in the gut, and ultimately alleviated constipation and potentially depressive symptoms [108]. Another study showed that early-life diets containing bioactive milk fractions and prebiotics could reduce anxiety-related behavior, and increase *Lactobacillus* spp. in juvenile rats, which was positively correlated with changes in serotonin (5-HT)1A and 5-HT2C mRNA expression [87]. It was reported that *Lactiplantibacillus plantarum* ST-III-fermented milk improved the autistic-like behaviors in male ASD mice through modulating specific gut microbes, such as increasing the relative abundance of family *Lachnospiraceae* and genus *Kineothrix* [88]. In addition, the mixture of almond baru (*Dipteryx alata* Vog.) and goat whey modulated gut microbiota (e.g., reducing the pathogenic genus *Clostridia_UCG-014*) improved memory and relieved anxiety in elderly rats [89]. Moreover, compared with the non-fermented control beverage, fermented dairy beverage increased the abundance of *Lactobacillus* by 235%, decreased *Phascolarctobacterium* by 25% in gut microbiota, and improved hippocampal function [107]. However, another large-scale study found no significant link between habitual yoghurt consumption and mental status improvement, and even high frequency of yoghurt consumption (≥twice/day) was related to increased depressive symptoms [118]. This discrepancy might be associated with lots of sugars or artificial sweeteners in many commercially available yoghurts, which could affect the results. Therefore, yoghurt products for patients with depression should be chosen carefully.

To sum up, dairy products and their bioactive components showed neuroprotective effects via modulation of gut microbiota (Figure 2, Table 3 and Table 4). Since fermented dairy beverages could contain some probiotics and prebiotics, more fermented dairy products should be investigated and developed with different beneficial bacteria, and more attention needs to be given to their influence on mental disorders. Moreover, because the studies that directly examine the link between dairy consumption and mental health are still limited, more clinical trials are needed to demonstrate their roles in mental disorders. 

### 3.4. Spices

Spices have a long history of being used as both food flavorings and traditional medicine, which possess many bioactive functions, such as anti-inflammatory, antibacterial, antifungal, and anticancer activities [119,120]. Moreover, the preventive and therapeutic effects of spices on mental disorders have received increasing attention. Curcumin, the principal bioactive compound in turmeric (*Curcuma longa*), shows various biological activities [121,122]. It was reported that curcumin could ameliorate dextran sulfate sodium salt (DSS)-induced anxiety-like behaviors in mice via the microbial–gut–brain axis. The study further indicated that curcumin partly reversed DSS-induced changes in gut microbiota [90]. In addition, a study conducted on mice indicated that capsaicin, the main bioactive component in chili peppers (*Capsicum annuum* L.), alleviated lipopolysaccharide-induced depressive-like behaviors and reduced levels of 5-HT and TNF-α by enhancing the relative abundances of certain gut microbiota, such as *Ruminococcus* and *Prevotella* [91]. Furthermore, a study found that the volatile oil of *Zanthoxylum bungeanum* may have a significant impact in alleviating and mitigating symptoms of depression via restoring the chronic unpredictable stress-induced gut microbiota dysbiosis, such as increasing Bacteroidales_S24-7_group, Lactobacillaceae, and Prevotellaceae, and decreasing Lachnospiraceae [92].

Collectively, spices and their components (such as *Zanthoxylum bungeanum*, curcumin, and capsaicin) showed protective effects against anxiety and depression via regulating gut microbiota (Figure 2 and Table 3). More spices and their bioactive compounds deserve further investigation for their role in the prevention and treatment of different mental disorders by regulating intestinal bacteria, which are at least partly associated with their powerful antibacterial properties.

### 3.5. Other Natural Products

Many other natural products also played vital roles in the prevention and management of mental disorders through the regulation of gut microbiota, such as fruits, vegetables, and medicinal herbs. A study showed that a higher intake of fruits and vegetables was positively correlated with better mental health based on 5845 Australian adults [123]. Another cross-sectional study showed that the intake of fruits and vegetables was inversely associated with inattention severity in ADHD children [124]. It was reported that after 8 weeks of administering flavonoid-rich orange juice to patients with depression, researchers found a significant reduction in depression scores and increased abundances of *Lachnospiraceae_uc* and *Bifidobacterium_uc* [109]. Additionally, a double-blind RCT showed that Cereboost^®^ (American ginseng extract) enhanced memory and attention via regulating gut microbiota. The study further found that Cereboost^®^ could increase levels of certain SCFAs in the intestinal tract through increasing the abundances of *Akkermansia muciniphila* and *Lactobacillus* by an in vitro model [93]. Another study found that one water-soluble polysaccharide from *Ginkgo biloba* leaves could reduce stress-induced depression through reversing gut dysbiosis [97]. Moreover, a study showed that the intake of *Lycium barbarum* polysaccharide during pregnancy could reduce the emotional injury of offspring caused by prenatal chronic stress through modulating the intestinal microbiota, such as enhancing the diversity of gut microbiota [94]. Additionally, a study conducted on mice showed that Chaihu-Shugan-San (a mixture of several medicinal herbs) alleviated restraint stress-generated anxiety and depression through inducing NF-κB-involved BDNF expression via reversing the restraint stress-induced changes in gut microbiota [95].

**Figure 3:**
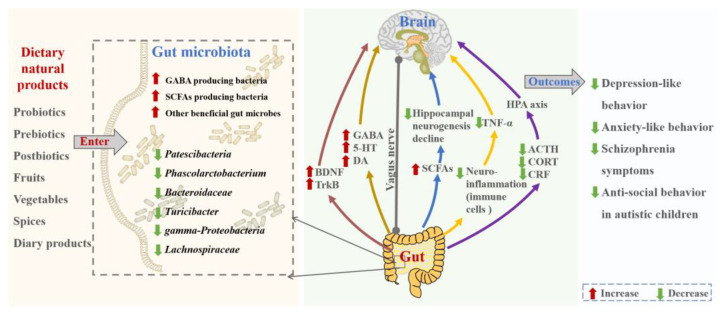
The effects and mechanisms of natural dietary products on mental disorders by targeting gut microbiota. Abbreviations: ACTH, adrenocorticotropic hormone; BDNF, brain-derived neurotrophic factor; CORT, cortisol; CRF, corticotropin releasing factor; DA, dopamine; GABA, glutamate–glutamine–gamma-aminobutyric acid; HPA, hypothalamic–pituitary–adrenal; SCFAs, short-chain fatty acids; TrkB, tyrosine kinase receptor B; TNF-α, tumor necrosis factor alpha; and 5-HT, 5-hydroxytryptamine.

In a word, several fruits, vegetables, and medicinal herbs could alleviate the severity of mental disorders and enhance mental health via modulating the gut microbiota (Figure 3). In the future, it is important to conduct further research to explore the effects of various fruits, vegetables, and medicinal herbs, as well as their bioactive components on different mental disorders. Additionally, tea (*Camellia sinensis*, such as green tea and black tea) and tea-like beverages (non-*Camellia sinensis* tea, such as vine tea and sweet tea) should be given increased attention, because they are popular beverages with many bioactivities and beneficial effects.

## 4. Conclusions

The gut microbiota and its metabolites could play an important role in mental health through the microbiota–gut–brain axis. The composition and abundance of gut microbiota, especially Firmicutes and Bacteroidetes, were associated with several mental disorders, such as anxiety, depression, bipolar disorder, ASD, and schizophrenia. At present, most studies about gut microbiota with mental disorders focused on the genus level, and more studies on gut microbiota should be carried out at the species level in the future, because the different species in the same genera could have different effects (even the opposite function) on mental disorders. Furthermore, due to the potential significant differences in the composition of the gut microbiome among individuals, it is crucial to accurately identify the changes of featured microbes that occur in each individual with mental disorders, which is important for personalized treatment of mental disorders through targeting gut microbiota. The epidemiological, experimental, and clinical studies have revealed that many kinds of probiotics (particularly *Lactobacillus* and *Bifidobacterium*), prebiotics (e.g., dietary fiber, GOS, B-GOS^®^, and alpha-lactalbumin), synbiotics, postbiotics (e.g., SCFAs), dairy products, spices (e.g., *Zanthoxylum bungeanum*, curcumin, and capsaicin), fruits, vegetables, and medicinal herbs could prevent and manage the mental disorders by modulating intestinal microbiota, including increasing beneficial gut microbiota and reducing harmful gut microbiota. Therefore, the supplement of dietary components mentioned above could be potential prevention and treatment strategies for mental disorders, except pharmacotherapy and psychotherapy. In addition, when the results from animal experiments are extrapolated to human beings, the differences between animals and humans should be considered. In the future, a greater number of experimental studies and high-quality large-sample clinical trials are required to explore the effects of more dietary components on mental disorders through the microbiota–gut–brain axis, and synbiotics and postbiotics need highlighting. Meanwhile, further elucidation and investigation of the underlying mechanisms of action is imperative. This paper is helpful for the public to choose natural dietary products to maintain mental health, and for natural dietary products to be developed into pharmaceuticals and functional foods for the prevention and treatment of several mental disorders.

## Figures and Tables

**Table 1:** The relationships between gut microbiota and mental disorders from epidemiological studies.

Study Type	Participants	Changes of Gut Microbiota in Mental Diseases	Ref.
Anxiety			
Case-control study	14 adult individuals with social exclusion and 25 HCs	↓ *Firmicutes/Bacteroidetes* and *Faecalibacterium* spp.	[30]
Prospective observational study	129 patients with active UC, 49 patients with depression and anxiety (non-UC), and 62 HCs	↓ *Prevotella_9*, *Lachnospira* ↑ *Lactobacillales*, *Sellimonas*, and *Streptococcus*	[31]
Cross-sectional study	40 GAD patients and 36 HCs	↑ *Fusobacteria*, *Bacteroidetes* spp. ↓ *Firmicutes* spp., *Lachnospira*, and *Butyricicoccus*	[32]
Depression			
Cross-sectional study	24 CDE patients and 16 HCs	↑ *Akkermansia*, *Clostridium_sensu_stricto_1*, *UBA1819* ↓ *Dialister*, *Fusicatenibacter*, and *Lachnospira*	[33]
Cohort study	1054 samples	↓ *Dialister*, *Coprococcus* spp.	[34]
Cross-sectional study	60 MDD patients and 60 HCs	↑ phylum Firmicutes, genera *Prevotella* and *Klebsiella* ↓ *Bacteroidetes*	[35]
Cross-sectional study	10 MDD patients and 10 HCs	↑ Firmicutes, Actinobacteria ↓ Bacteroidetes, Proteobacteria	[36]
-	91 premenopausal females with depression and 98 HCs	↑ *Klebsiella aerogenes*	[37]
Bipolar disorder			
Cross-sectional study	23 BD patients and 23 HCs	↑ *Clostridiaceae* and *Collinsella*	[38]
Cross-sectional study	115 BD patients and 64 HCs	↓ *Faecalibacterium*	[39]
Cross-sectional study	32 BD patients and 10 HCs	↑ phylum Actinobacteria and Coriobacteria ↓ *Ruminococcaceae* and *Faecalibacterium*	[40]
Cross-sectional study	113 BD patients, 39 unaffected first-degree relatives, and 77 HCs	↑ *Flavonifractor*	[41]
Autism spectrum disorder			
Cross-sectional study	39 PTHS children and 46 unaffected family members	↑ *Clostridium bolteae*	[42]
Cross-sectional case-control study	ASD children with (*n* = 60) or without (*n* = 60) sleep disorder	↓ *Faecalibacterium* and *Agathobacter*	[43]
Cross-sectional study	30 ASD children and 30 neurotypical controls	↑ *Clostridium paraputri*, *Clostridium bolteae*, and *Clostridium perfringens*	[44]
Cross-sectional case-control study	48 ASD children and 57 HCs	↑ *Actinobacteria, Proteobacteria and Bacilli*	[45]
Schizophrenia			
Cross-sectional study	38 schizophrenia patients and 20 HCs	↓ *Acetanaerobacterium*, *Haemophilus*, and *Turicibacter*	[46]
Cross-sectional study	42 patients with acute schizophrenia, 40 patients with schizophrenia in remission, and 44 HCs	↑ *Haemophilus* ↓ *Coprococcus*	[47]
Cross-sectional study	38 schizophrenia patients and 38 NCs	↑ *Veillonella* ↓ *Ruminococcus* and *Roseburia*	[48]
Cross-sectional study	48 schizophrenia patients and 48 NCs	↑ *Lachnospiraceae*	[49]
Cross-sectional study	82 schizophrenia patients and 80 NCs	↑ *Collinsella, Lactobacillus, and Succinivibrio* ↓ *Adlercreutzia, Anaerostipes, and Ruminococcus*	[50]
Cross-sectional study	10 schizophrenia patients and 16 HCs	↑ *Proteobacteria* ↓ *Faecalibacterium* and *Lachnospiraceae*	[51]
Metagenome-wide association study	90 medication-free schizophrenia patients and 81 NCs	↑ *Lactobacillus fermentum, Enterococcus faecium*, and *Alkaliphilus oremlandii*	[52]
Anorexia nervosa			
Cohort study	77 females with anorexia nervosa and 70 HCs	↑ *Erysipelatoclostridium ramosum*, *Enterocloster bolteae* ↓ *Eisenbergiella*, butyrate-producing bacterium	[53]
Posttraumatic stress disorder			
Longitudinal investigation	71 FHWs and 104 SHWs	↑ *Bacteroides eggerthii* ↓ *Eubacterium hallii* group uncultured bacterium	[54]
Case-control study	79 PTSD participants and 58 TECs	↑ *Mitsuokella*, *Odoribacter*, and *Catenibacterium*	[55]
Attention deficit hyperactivity disorder			
Case-control study	41 ADHD children and 39 HCs	↑ *Agathobacter*, *Anaerostipes*, and *Lachnospiraceae*	[56]
Others			
Cross-sectional study	198 individuals	Anxiety: ↓ Simpson’s diversity PTSD, depression, and trait anxiety: ↓ *Fusicatenibacter saccharivorans*	[57]

↑ represents positive association, ↓ represents negative association. Abbreviations: ADHD, attention deficit hyperactivity disorder; ASD, autism spectrum disorders; BD, bipolar disorder; CDE, current depressive episode; FHWs, frontline healthcare workers; GAD, generalized anxiety; HCs, healthy controls; MDD, major depressive disorder; NCs, normal controls; SHWs, second-line healthcare workers; TECs, trauma-exposed controls; UC, ulcerative colitis.

**Table 2:** The relationships of gut microbiota and mental disorders from experimental studies.

Study Type	Animals	Changes of Gut Microbiota in Mental Diseases	Ref.
Anxiety			
In vivo	BALB/c, Orient C57BL/6N, Taconic C57BL/6N, and Taconic C57BL/6J mice	↓ Firmicutes	[58]
Depression			
In vivo	Female depression-like macaques	↑ family Paraprevotella ↓ families Streptococcaceae, Gemella	[59]
In vivo	C57BL/6J female mice	↑ *Klebsiella aerogenes*	[37]
Autism spectrum disorder			
In vivo	*Fmr1* KO mice on a C57BL/6J background	↓ *Akkermansia muciniphila*	[60]

↑ represents positive association, ↓ represents negative association.

**Table 3:** The effects of dietary components on mental disorders from experimental studies.

Study Type	Subjects	Methods	Alterations of the Gut Microbiota	Ref.
Probiotics				
In vivo	Male Sprague–Dawley rats	*L. casei* for 3 weeks	↑ *Blautia* and *Roseburia* ↓ Prevotella	[78]
In vivo	Male C57BL/6 mice	*L*. *rhamnosus* zz-1 for 6 weeks	↑ *Lachnospiraceae* NK4A136 group, *Bacteroides*, and *Muribaculum*	[79]
In vivo	Male C57BL/6 J mice	Diet supplemented with 0.125% heat-killed EC-12	↑ *Butyricicoccus* and *Enterococcus*	[80]
In vivo	Male ICR mice	Mixture of *L. plantarum* LP3, *L. rhamnosus* LR5, *B. lactis* BL3, *B. breve* BR3, and *P. pentosaceus* PP1 for 8 weeks	↑ *Actinobacteria*, *Cyanobacteria*, and S24-7_unclassified	[81]
In vivo	Sprague–Dawley rats	Mixture of *B. coagulans* unique IS-2, *L. plantarum* UBLP-40, *L. rhamnosus* UBLR-58, *B. lactis* UBBLa-70, *B. breve* UBBr-01, and *B. infantis* UBBI-01 for 6 weeks	↑ *Firmicutes*/*Bacteroides*	[82]
In vivo	Male C57BL/6 mice	*Pediococcus acidilactici* CCFM6432 for 5 weeks	↑ Bifidobacterium ↓ *Escherichia-shigella*	[83]
In vivo	Male C57BL/6 mice	*Akkermansia muciniphila* for 3 weeks	↑ *Verrucomicrobia* and *Akkermansia* ↓ *Helicobacter*, *Lachnoclostridium, and Candidatus_Saccharimonas*	[84]
Prebiotics and postbiotics				
In vivo	Female C57BL/6J mice	Inulin (37 g/1000 kcal) for 20 weeks	↑ *Lactobacillus*, *Prevotella*, and *Lactobacillus*	[85]
In vivo	Male BALB/c mice	SCFA-acylated starches (20 *w*/*v*) for 3 weeks	↑ *Odoribacter* and *Oscillibacter*	[86]
Dairy products				
In vivo	Juvenile (PND 24), male Fischer 344 rats	GOS (21.23 g/kg), PDX (6.58 g/kg), lactoferrin (1.86 g/kg) and whey protein concentrate MFGM-10 (15.9 g/kg)	↑ *Lactobacillus* spp.	[87]
In vivo	ICR mice	400 μL *L. plantarum* ST-III-fermented milk in the morning and evening for 2 weeks	↑ family *Lachnospiraceae* and genus *Kineothrix*	[88]
In vivo	40 elderly male Wistar rats	2000 mg of almond baru+ 2000 mg of goat milk whey/kg for 10 weeks	↑ *Gastranaerophilales and Ruminococcaceae* ↓ *Clostridia_UCG-014*	[89]
Spices				
In vivo	C57BL/6 mice	Curcumin (100 mg/kg/d) for 8 d	↑ *Bacteroidetes*, *Muribaculaceae_unclassified* ↓ *Deinococcus-Thermus*, *Bacteroides*, and *Ruminococcaceae_unclassified*	[90]
In vivo	Male C57BL/6 mice	Diet supplement with 0.005% capsaicin for 4 months	↑ *Ruminococcus*, *Prevotella*, and *Allobaculum*	[91]
In vivo	Male Sprague–Dawley rats	VOZB (50, 100 and 200 mg/kg/d) by an intragastric gavage for 14 d	↑ Bacteroidales_S24-7_group, Lactobacillaceae, and Prevotellaceae ↓ *Lachnospiraceae*	[92]
Medicinal herbs				
In vitro	Simulator of the human intestinal microbial ecosystem	Cereboost^®^ (200 mg/d) for 3 weeks	↑ *Lactobacillus* and *Akkermansia muciniphila*	[93]
In vivo	Sprague–Dawley rats	LBP (40 mg/kg) for 14 d	↑ *Firmicutes* ↓ *Turicibacter*	[94]
In vivo	Male C57BL/6 mice	CSS (1.0 g/kg/d) for 5 d	↑ *Lactobacillaceae*, *Prevotellaceae*, and *AC160630_f* ↓ gamma-*Proteobacteria*	[95]
In vivo	Male C57BL/6 mice	Xiaoyaosan (0.658 g/kg/d) for 14 d	↑ *Lachnospiraceae* ↓ *Bacteroidaceae*	[96]
In vivo	Male BALB/c mice	GPS (300 mg/kg in PBS) for 4 weeks	↑ *Prevotellaceae*, *Erysipelotrichaceae*, and *Family_*XIII	[97]

↑ represents increase, ↓ represents decrease. Abbreviations: CFU, colony-forming unit; CSS, Chaihu-Shugan-San; EC-12, *Enterococcus faecalis* strain EC-12; GOS, galacto-oligosaccharides; GPS, water-soluble polysaccharide from *Ginkgo biloba* leaves; *L*. *casei*, *Lactobacillus casei*; LBP, *Lycium barbarum* polysaccharide; *L. plantarum, Lactobacillus plantarum*; *L. rhamnosus*, *Lactobacillus rhamnosus*; PND, postnatal day; PDX, polydextrose; *P. pentosaceus, Pediococcus pentosaceus*; and VOZB, volatile oil of *Zanthoxylum bungeanum*.

**Table 4:** The effects of dietary components on mental disorders from clinical trials.

Study Type	Participants	Methods	Alterations of the Gut Microbiota	Ref.
Probiotics				
Double-blind placebo RCT	12 postgraduate student volunteers	*L*. *rhamnosus* Probio-M9 for 21 d	↑ *Barnesiella* and *Akkermansia*	[98]
Double-blind placebo RCT	156 healthy adults with subclinical symptoms of depression, anxiety, and insomnia	Two 500 mg capsules for 8 weeks	↑ *Bifidobacteriaceae* and *Lactobacillacea* ↓ *Enterobacteriaceae*	[99]
Double-blind placebo RCT	103 stressed adults	*L. plantarum* P-8 for 12 weeks	↑ *Bifidobacterium adolescentis*, *Bifidobacterium longum* and *Fecalibacterium prausnitzii* ↓ *Roseburia faecis* and *Fusicatenibacter saccharivorans*	[100]
Double-blind placebo RCT	45 patients with MDD	*Bifidobacterium breve* CCFM1025 powder for 4 weeks	↑ *Desulfovibrio* and *Faecalibaculum*	[101]
Monocentric, placebo RCT	82 depressed individuals	Mixture of 9 probiotics for 28 d	↓ *Ruminococcus gauvreauii* and *Coprococcus 3*	[102]
Prebiotics and postbiotics				
Double-blind placebo RCT	64 healthy females	GOS prebiotic for 28 d	↑ *Bifidobacterium*	[103]
Double-blind placebo RCT	33 healthy subjects	Natural multi-ingredient targeted mental wellness supplement for 4 weeks	↑ *Lactobacillus* and *Bifidobacterium*	[104]
Double-blind placebo RCT	30 autistic children	B-GOS^®^ for 6 weeks	↑ *Coprococcus* spp., *Dorea formicigenerans*, and *Oribacterium* spp.	[105]
Double-blind placebo RCT	22 patients with depression	LS for 24 weeks	No significant change	[106]
Dairy products				
Cross-over RCT	26 healthy adults	A dairy-based fermented beverage for 4 weeks	↑ *Lactobacillus* ↓ *Phascolarctobacterium*	[107]
Double-blind placebo RCT	82 depressive patients with constipation	Fermented dairy beverage for 9 weeks	↑ *Adlercreutzia*, *Megasphaera*, and *Veillonella* ↓ *Rikenellaceae_RC9_gut_group*, *Sutterella*, and *Oscillibacter*	[108]
Fruits				
RCT	40 participants	Flavonoid-rich orange juice for 8 weeks	↑ *Lachnospiraceae_uc*, *Bifidobacterium_uc*, and *Eubacterium_g4*	[109]

↑ represents increase, ↓ represents decrease. Abbreviations: *B. adolescentis*, *Bifidobacterium adolescentis*; B-GOS^®^, Bimuno^®^ galactooligosaccharide; GOS, galacto-oligosaccharides; *L. casei*, *Lactobacillus casei*; *L. plantarum*, *Lactobacillus plantarum*; *L. reuteri*, *Lactobacillus reuteri*; LS, 4G-beta-D-Galactosucrose; and RCT, randomized controlled trial.

## Data Availability

Not applicable.
